# Cognitive Assessment of Older People: Do Sensory Function and Frailty Matter?

**DOI:** 10.3390/ijerph16040662

**Published:** 2019-02-24

**Authors:** Ruby Yu, Jean Woo

**Affiliations:** 1 Jockey Club Institute of Ageing, The Chinese University of Hong Kong, Hong Kong, China; jeanwoowong@cuhk.edu.hk; 2 Department of Medicine and Therapeutics, Faculty of Medicine, The Chinese University of Hong Kong, Hong Kong, China

**Keywords:** cognitive impairment, cognitive assessment, sensory function, vision, hearing, frailty, ageing

## Abstract

*Background*: To examine the associations of visual and hearing functions, and frailty with subjective memory complaints (SMCs) in a community primary care pilot project of older people aged 60 years and over. *Methods*: The study was conducted in 24 community centers. A total of 1949 community-dwelling older people aged between 60–97 years were evaluated for which detailed information regarding socio-demographics, lifestyle, and clinical factors were documented at baseline and an average of 12 months later. SMCs were assessed using the 5-item Abbreviated Memory Inventory for the Chinese (AMIC). Visual and hearing functions were measured with two separate single questions. Frailty was assessed using a simple frailty question (FRAIL). *Results*: At baseline, 1685 (74.6%) participants had reported at least 3 SMCs (AMIC score ≥ 3). Of the 573 participants without / with 1–2 SMCs (AMIC score = 0–2) at baseline, 75 had incomplete data regarding SMCs and 190 developed at least 3 SMCs after 12 months. After adjustments for age, sex, marital status, educational level, hypertension, and diabetes at baseline, poor vision (OR 2.2 95% CI 1.8–2.7), poor hearing (OR 2.2 95% CI 1.8–2.8), and frailty (OR 4.6 95% CI 3.1–6.7) at baseline were each significantly associated with an increased risk of at least 3 SMCs at follow-up. After a further adjustment for baseline SMCs, the associations remained significant. Similar results were obtained when incident SMCs and improvement in subjective memory were used as the outcome variables; and *Conclusions*: In the care of older people, detection of sensory impairment and frailty through screening may allow formulation of strategies to prevent or delay the onset of cognitive decline.

## 1. Introduction

Alzheimer’s disease (AD) and other types of dementia affect older adults globally. People with mild cognitive impairment (MCI), especially MCI involving memory problems, have an increased risk of developing Alzheimer’s disease (AD), with an annual progression rate of 10%–20% [1]. However, neuropathological changes of AD may occur many years before the onset of cognitive impairment or MCI [2,3,4,5]. Therefore, identifying early symptoms of cognitive decline can lead to the timely detection of diseases that lead to dementia, allowing for improved care and planning.

Subjective memory complaints (SMCs) have been suggested as a clinical indicator of cognitive decline and impairment before AD or other neurodegenerative processes in the course of the clinical manifestation of AD [6]. Short screening tests (e.g., Abbreviated Memory Inventory for Chinese, AMIC [7]) or simple questions (e.g., “Do you feel like your memory is becoming worse?” [6]) have been used to screen for SMCs in predicting cognitive outcomes. Longitudinal studies among older people without cognitive impairment identified SMCs as a predictor of cognitive decline and dementia [6,8,9,10,11,12,13]. A meta-analysis of 28 studies also demonstrated that older people with SMCs are more likely to develop dementia than those without SMCs [14] Nevertheless, SMCs may be particularly predictive for subsequent cognitive decline when they are accompanied by sensory impairment and/or frailty. A recent study suggests an association exists between sensory impairment and SMCs [15]. In addition, there is growing evidence to show the importance of sensory functions and frailty status for cognitive outcomes. Numerous longitudinal studies have demonstrated associations of visual and hearing impairments with cognitive decline [16,17,18,19,20], although conflicting findings have also been reported in another study [21]. Frailty, a state of decline in function reserves [22], has been associated with cognitive decline and subsequently major neurodegenerative disorder (dementia) [23,24,25,26,27,28]. Despite sensory function and frailty predicting cognitive decline, less attention has been paid to the inclusion of these conditions in cognitive assessment.

Therefore, we examined the associations of visual and hearing function and frailty with SMCs in a community primary care pilot project of older people aged 60 years and over where SMCs was assessed over a duration of 12 months. If visual and hearing functions and frailty are associated with SMCs/predictive of the development of SMCs, the implication is that, these conditions should form an important part of cognitive assessment.

## 2. Materials and Methods

### 2.1. Setting and Participants

A community pilot project on a model of primary care of older people was started in 2016, based in 24 community centers in all three regions of Hong Kong (Hong Kong Island, Kowloon, and the New Territories). The primary aim was to improve community care of older people with hypertension and diabetes. Fifty members of each center aged 60 years and over were invited to participate. Priority was given to those with known hypertension and diabetes, and those judged by the center members to benefit from community group activities. The study was approved by the Survey and Behavioral Ethics Committee of the Chinese University of Hong Kong (No. 126-16). Informed consent was obtained from all participants.

### 2.2. Data Collection

A brief multi-domain geriatric assessment was used to screen for geriatric conditions. It was self-administered electronically using an e-tablet. Screening results were uploaded to a secure server for subsequent analyses. Positive responses were flagged and a report was generated to project team members, in order to identify issues to be followed-up with shared decision making on what pathways (i.e., interventions and courses of action) to pursue. Resources to guide interventions and actions for each domain were compiled in conjunction with health professionals, using a stepped care approach of self-management, brief interventions and/or group-based support activities, and secondary care services. The brief geriatric assessment was repeated approximately 12 months later.

### 2.3. The Brief Geriatric Assessment

#### 2.3.1. Subjective Memory Complaints

The 5-item AMIC was used for screening for SMCs. AMIC is an abbreviated version of the original 27-item Memory Inventory for the Chinese (validated for the diagnosis of MCI, with a sensitivity of 54.6%–65.3% and specificity of 57.4%). There are five items: “Do you always forget where you put your stuff?”, “Do you feel that your memory is worse than your peers?”, “Do you always forget what you want to say during a conversation?”, “Do you always fail to find the appropriate word to express your idea during a conversation?”, and “Do you forget your acquaintances’ names when you meet them?”. AMIC scores range from 0–5 (1 point for each item; 0—best to 5—worse). An AMIC score ≥ 3 is predictive of MCI [6].

#### 2.3.2. Sensory Function

Visual and hearing functions were measured with two separate single questions, “Do you have any difficulty seeing things?” and “Do you have any difficulty hearing?”, respectively. The response categories were “very good”, “good”, “fair”, “not too well”, “poor”, and “very poor” which were collapsed to form a binary attribute for regression analysis (i.e., “very good” and “good” = “robust”; “fair”, “not too well”, “poor”, and “very poor” = “poor”).

#### 2.3.3. Frailty

Frailty was assessed using a simple frailty questionnaire (FRAIL). There are five items of the FRAIL scale: fatigue, resistance, ambulation, illnesses, and loss of weight. The FRAIL scores range from 0–5, with 0–1 point for each item and a score of 0 represents robust, 1–2 as pre-frail, and 3–5 as frail [29,30].

#### 2.3.4. Other Geriatric Symptoms

Other measures included a single question for chewing difficulties, a simple questionnaire to rapidly diagnose sarcopenia based on Strength, Assistance in walking, Rise from a chair, Climb stairs, and Falls (SARC-F), a single question for self-rated health, “How would you rate your general health?”, three questions for subjective well-being (life satisfaction, feelings of happiness, and sense of purpose and meaning in life), a single question for incontinence, “Do you have problems with incontinence?”, five items for Instrumental Activity Daily Living (IADL) impairments, a single question for medication, “How many types of medication prescribed by the doctor are you taking?”, and a single question for finance issue, “Do you have enough money for day to day use?”. However, these measures were not included in the analysis.

### 2.4. Statistical Analysis

Data were summarized as means (standard deviations) for continuous variables and as percentages for categorical data. Participant characteristics were compared across cognitive status at baseline based on the AMIC score (No SMC with an AMIC score = 0; 1–2 SMCs with an AMIC score = 1–2, at least 3 SMCs with an AMIC score ≥ 3) using analysis of variance (ANOVA) for continuous variables and chi-square tests for categorical variables. Logistic regression models were used to estimate the longitudinal associations of visual function (robust, poor), hearing function (robust, poor), and frailty status (robust, pre-frail, and frail) at baseline and SMCs after an average of 12 months. Progressive models were used as follows: model 1, no adjustment; model 2, adjustment for age and sex; model 3, further adjustments for marital status (not married, married) and educational level (no education/primary, secondary/tertiary); and model 4 further adjustments for hypertension (no, yes) and diabetes (no, yes). An additional model (Model 5) was performed with a further adjustment for baseline SMCs. Sensitivity analyses were also performed to see how redefining the outcome variable (using incident SMCs and improvement in subjective memory) changes the observed effects of visual and hearing functions, and frailty on SMCs. Analyses were carried out using the Window-based SPSS Statistical Package v24.0 (IBM Corp. Released 2013. Armonk, NY, USA), and *p* values less than 0.05 were considered statistically significant.

## 3. Results

### 3.1. Baseline Characteristics of the Study Population

At baseline, 2259 Chinese people aged between 60–97 years completed the brief geriatric assessment. The mean age of the participants was 76.1 years, 76.9% were women, 47% were married, 72% received at least primary education, and 34.9% were living alone. In total, 74.6% had at least 3 SMCs, 57.8% and 44.3% reported poor vision and poor hearing, respectively and additionally, 64.2% had a FRAIL score ≥ 1 (i.e., classified as pre-frail and frail) (Table 1).

### 3.2. Factors Associated with SMCs

As expected, participants who had at least 3 SMCs were older, predominantly women (81.1%), and more likely to be living alone (36.6%), and with higher proportions of poor vision (64.0%), poor hearing (49.6%), and frailty (71.7%). They also had a lower proportion of being married (45.4%) and a lower level of education (secondary education or above, 23.9%) (Table 2).

### 3.3. Longitudinal Associations of Poor Vision, Poor Hearing, and Frailty with SMCs

**Figure 1:**
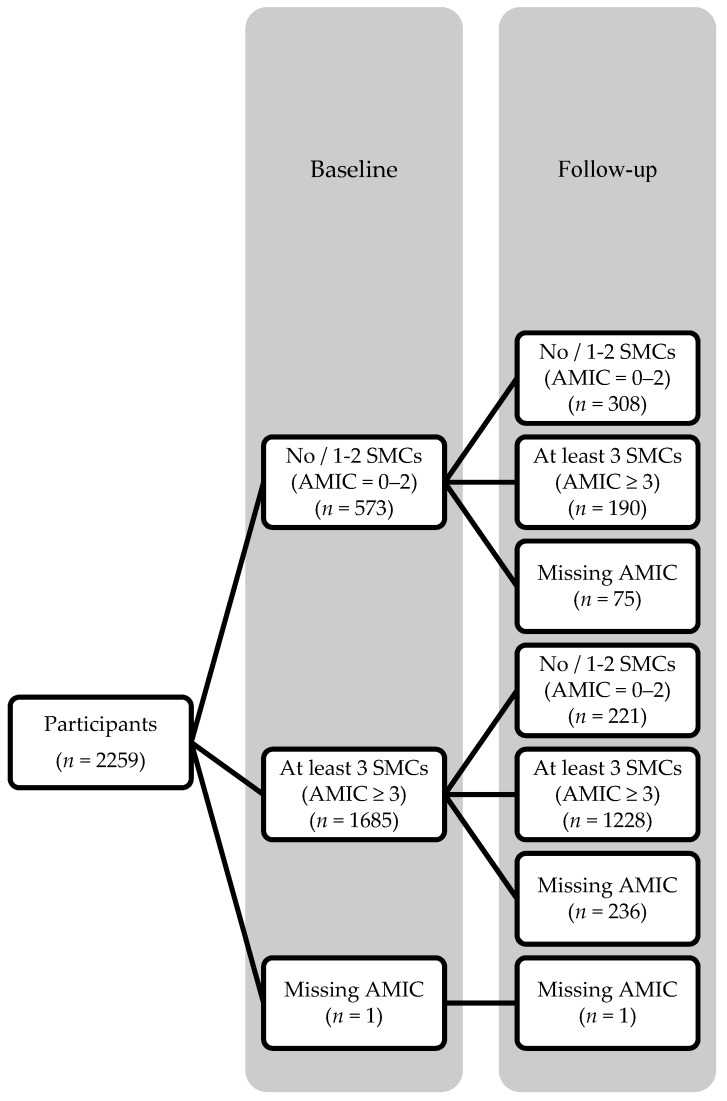
Flow chart of participants.

An average of 12 months after baseline, 1949 participants returned with 1947 completed the 5-item AMIC and 1418 (72.8%) participants had at least 3 SMCs. Of the 498 participants without / with 1–2 SMCs at baseline that returned at follow-up, 190 (38.2%) had developed at least 3 SMCs at follow-up (Figure 1).

Poor vision (OR 2.2 95% CI 1.8–2.7), poor hearing (OR 2.2 95% CI 1.8–2.8), and frailty (OR 4.6 95% CI 3.1–6.7) at baseline were each significantly associated with an increased risk of SMCs (AMIC score ≥ 3) at follow-up, after adjustment for age, sex, marital status, educational level, hypertension, and diabetes at baseline. After a further adjustment for baseline SMCs, the associations remained significant (Table 3).

Sensitivity analyses were performed using incident SMCs and improvement in subjective memory as the outcome variables. Of the 498 participants without / with 1–2 SMCs at baseline, poor vision (OR 1.6, 95% CI 1.1–2.3), poor hearing (OR 1.9, 95% CI 1.2–2.8), and frailty (OR 3.1, 95% CI 1.5–6.8) were each significantly associated with an increased risk of incidence of SMCs (AMIC score ≥ 3), independent of age, sex, marital status, educational level, hypertension, and diabetes at baseline (Table 4). Of the 1449 participants with at least 3 SMCs at baseline that returned at follow-up, robust vision (OR 1.6, 95% CI 1.2–2.2), robust hearing (OR 1.5, 95% CI 1.1–2.1), robust (OR 2.6, 95% CI 1.6–4.2) and pre-frailty (OR 2.0, 95% CI 1.2–3.1) status were each significantly associated with an increased likelihood of improvement in subjective memory, independent of age, sex, marital status, educational level, hypertension, and diabetes at baseline (Table 5).

## 4. Discussion

In this community-based study, the proportion of people with SMCs was high. Moreover, participants with poor vision, poor hearing, or frailty at baseline were associated with an increased risk of SMCs after an average of 12 months, independent of age, sex, marital status, educational level, hypertension, diabetes at baseline. These findings reinforce the importance of assessing sensory function and frailty status in cognitive assessment of older people.

Our findings indicate that visual and hearing functions were significant and independent predictors of SMCs among older people aged 60 and over. This finding is in concordance with the observation from a national survey in the United Kingdom showing a significant proportion of visual impairment (approximately one-third) detected in a sample of people with dementia [31]. A recent pooled analysis of data from eleven studies reported that individuals with either peripheral or central hearing dysfunction had a higher risk of having cognitive impairment at follow-up [19]. Hence, our results add further support to the possibility that sensory impairment may be an early symptom of an underlying neurodegenerative process (such as degeneration of central nervous structures), before the clinical manifestation of cognitive impairment. Poor visual and hearing functions may also contribute to reduced physical and mental activities (indicating a prolonged lack of adequate sensory input), which in turn predisposes to cognitive deterioration possibility due to neuronal atrophy [32]. Nevertheless, there may be other reasons for an older person with sensory impairment that leads to cognitive decline. For example, older people who cannot see well or hear well may become depressed, which increases the risk of cognitive deterioration. Although we did not collect information regarding depressive symptoms, analyses of our data found that poor vision and poor hearing were significantly associated with low subjective well-being (in terms of life satisfaction, feelings of happiness, and sense of purpose and meaning in life), which was positively associated with SMCs (data not shown). Our study could have potential public health implications, as it suggests that identifying and targeting sensory problems in older people could be a potentially useful strategy for preventing cognitive decline.

Our finding also demonstrates that frailty was a significant and independent predictor of SMCs. This finding extends the findings of previous studies that frailty predicts cognitive decline and incident dementia [23,24,25,26,27,28], and thus adds support to the possibility that frailty may predispose to cognitive decline and subsequently major neurodegenerative disorder (dementia). Although the underlying mechanisms are still unclear, a chronic inflammatory state has been postulated to be an underlying etiology of frailty and cognitive impairment [33]. An alternative explanation is that frailty may be causally associated with reduced physical and mental activities [34], which are related to an increased risk of cognitive decline [35]. While frailty predicts cognitive decline, some studies have suggested that cognitive impairment predicts frailty [36]. Furthermore, there is increasing evidence that, even with normal ageing, both frailty and cognitive decline often coexist [37], and the occurrence of both conditions has been associated with higher risks of adverse health outcomes [38]. Therefore, among older people, frailty may indicate the presence of cognitive decline, and should be part of cognitive assessment for older people in the community.

The significant longitudinal associations of poor vision, poor hearing, and frailty with SMCs provide evidence of their suitability for applications in the community settings. The WHO guidelines on community-level interventions to manage declines in intrinsic capacity (Integrated Care For Older People, ICOPE) also recommend assessing indicators of decline in physical and mental capacities, using a people-centered approach [39]. Therefore, simple screening questions (e.g., the presence of visual and hearing impairments) and frailty assessment (e.g., the FRAIL scale) can be adopted in community settings. Positive responses to the questions/assessment should trigger further assessments (e.g., visual acuity, hearing capacity, activity of daily living, etc.) and management by health support network in the community.

Our study should be viewed within the context of its limitations. First, the AMIC was used to screen for SMCs and as a proxy for measuring cognitive decline. Due to the lack of formal testing, there remains some uncertainty as to whether some of the participants classified as having SMCs might actually have had MCI. Second, the specificity of the AMIC was relatively low, as memory complaints were frequently reported among those with normal cognition [6]. Third, standardized tests of vision and hearing were not performed, which may introduce information bias. Nevertheless, the AMIC score and the simple questions used to screen for visual and hearing problems in this study help trigger further in-depth assessments, which may be used as the first step in a stepped-care approach to detect cognitive and sensory impairments in the community, allowing targeted intervention to potentially retard cognitive decline. Fourth, the follow-up period may not be long enough to capture the change in cognitive function. Fifth, data on unmeasured factors, such as comorbid depression which is an important risk factor for cognitive impairment, were not available and could not be controlled for. Lastly, although the findings of the present study are based on community-based sample obtained in 24 community centers in all three regions of Hong Kong, they may not be generalizable to institutionalized populations.

## 5. Conclusions

The findings of this study support the proposal that sensory function and frailty should be assessed together with cognitive assessment, which may enhance the prediction for cognitive decline and allow formulation of strategies to prevent or delay the onset of cognitive decline.

## Figures and Tables

**Table 1:** Baseline characteristics of the study population (*n* = 2259).

Variable	Mean ± SD/*n* (%)
Age (years), mean ± SD (range)	76.1 ± 7.4 (60–97)
Age, *n* (%)	
60–69 years	503 (22.3)
70–79 years	972 (43.0)
80+ years	784 (34.7)
Sex, *n* (%)	
Men	521 (23.1)
Women	1738 (76.9)
Marital status, *n* (%)	
Single	103 (4.6)
Married	1061 (47.0)
Widowed	958 (42.4)
Divorced/separated	137 (6.1)
Educational level, *n* (%) *	
No education	633 (28.0)
Primary	1002 (44.4)
Secondary	516 (22.9)
Tertiary	107 (4.7)
Living arrangement, *n* (%) *	
Living alone	788 (34.9)
Living with spouse	631 (27.9)
Living with children	482 (21.3)
Living with spouse and children	283 (12.5)
Living with others (e.g., domestic helper)	74 (3.3)
Self-reported hypertension, *n* (%)	
No	782 (34.6)
Yes	1477 (65.4)
Self-reported diabetes, *n* (%)	
No	1628 (72.1)
Yes	631 (27.9)
SMCs, *n* (%) *	
No SMC, AMIC score = 0	137 (6.1)
1–2 SMCs, AMIC score = 1–2	436 (19.3)
At least 3 SMCs, AMIC score ≥ 3	1685 (74.6)
Vision, *n* (%)	
Very good	346 (15.3)
Good	607 (26.9)
Fair	777 (34.4)
Not too well	391 (17.3)
Poor	127 (5.6)
Very poor	11 (0.5)
Hearing, *n* (%)	
Very good	599 (26.5)
Good	660 (29.2)
Fair	607 (26.9)
Not too well	302 (13.4)
Poor	83 (3.7)
Very poor	8 (0.4)
Frailty, *n* (%) *	
Robust, FRAIL score = 0	809 (35.8)
Pre-frail, FRAIL score = 1–2	1040 (46.1)
Frail, FRAIL score ≥ 3	409 (18.1)

Abbreviation: SMCs, Subjective Memory Complaints; AMIC, Abbreviated Memory Inventory for the Chinese. * Missing value: Educational level (*n* = 1); living arrangement (*n* = 1); SMCs (*n* = 1); frailty (*n* = 1). Percentages may not add up to 100% due to rounding.

**Table 2:** Baseline characteristics of the study population by cognitive status (*n* = 2258 *).

Variable	No SMC AMIC = 0	1–2 SMCs AMIC = 1–2	At Least 3 SMCs AMIC ≥ 3	*p*
	(*n* = 137)	(*n* = 436)	(*n* = 1685)	
Age, years	73.8 ± 7.4	75.6 ± 7.8	76.4 ± 7.3	<0.001
Women, %	78 (56.9)	293 (67.2)	1367 (81.1)	<0.001
Married, %	70 (51.1)	225 (51.6)	765 (45.4)	0.042
Secondary education or above, %	54 (39.4)	166 (38.2)	403 (23.9)	<0.001
Living alone, %	45 (33.1)	127 (29.1)	616 (36.6)	0.013
Hypertension, %	95 (69.3)	277 (63.5)	1105 (65.6)	0.441
Diabetes, %	44 (32.1)	116 (26.6)	471 (28.0)	0.456
Poor vision, %	43 (31.4)	184 (42.2)	1079 (64.0)	<0.001
Poor hearing, %	26 (19.0)	138 (31.7)	836 (49.6)	<0.001
Pre-frailty and frailty, %	46 (33.6)	204 (46.9)	1198 (71.1)	<0.001

Abbreviation: AMIC, Abbreviated Memory Inventory for the Chinese. * Missing data: *n* = 1.

**Table 3:** Associations of sensory impairment and frailty with subjective memory complaints (SMCs) after 12 months (*n* = 1947).

Variables	*n* (%)	Model 1		Model 2		Model 3		Model 4		Model 5	
		OR	95% CI	OR	95% CI	OR	95% CI	OR	95% CI	OR	95% CI
Vision											
Robust	816 (41.9)	Reference		Reference		Reference		Reference		Reference	
Poor	1131 (58.1)	2.3	1.9–2.8	2.3	1.8–2.8	2.2	1.8–2.8	2.2	1.8–2.7	1.6	1.3–2.0
Hearing											
Robust	1089 (55.9)	Reference		Reference		Reference		Reference		Reference	
Poor	858 (44.1)	2.2	1.7–2.7	2.2	1.8–2.7	2.2	1.8–2.8	2.2	1.8–2.8	1.7	1.3–2.1
Frailty											
Robust	713 (36.6)	Reference		Reference		Reference		Reference		Reference	
Pre-frail	902 (46.3)	1.9	1.6–2.4	1.9	1.5–2.3	1.9	1.5–2.3	1.9	1.5–2.3	1.3	1.0–1.7
Frail	332 (17.1)	4.8	3.3–6.9	4.5	3.1–6.6	4.6	3.1–6.7	4.6	3.1–6.7	2.7	1.8–4.1

Model 1: crude. Model 2: age and sex. Model 3: age, sex, marital status, and educational level. Model 4: age, sex, marital status, educational level, hypertension, and diabetes. Model 5: age, sex, marital status, educational level, hypertension, diabetes, and baseline cognitive status.

**Table 4:** Associations of sensory impairment and frailty with improvement in incident SMCs after 12 months (*n* = 498).

Variables	*n* (%)	Model 1		Model 2		Model 3		Model 4	
		OR	95% CI	OR	95% CI	OR	95% CI	OR	95% CI
Vision									
Robust	301 (60.4)	Reference		Reference		Reference		Reference	
Poor	197 (39.6)	1.6	1.1–2.4	1.6	1.1–2.3	1.6	1.1–2.3	1.6	1.1–2.3
Hearing									
Robust	356 (71.5)	Reference		Reference		Reference		Reference	
Poor	142 (28.5)	1.8	1.2–2.7	1.8	1.2–2.8	1.9	1.2–2.8	1.9	1.2–2.8
Frailty									
Robust	279 (56.0)	Reference		Reference		Reference		Reference	
Pre-frail	185 (37.1)	1.5	1.0–2.1	1.4	1.0–2.1	1.4	1.0–2.1	1.4	1.0–2.1
Frail	34 (6.8)	3.3	1.6–6.9	3.1	1.5–6.7	3.3	1.6–7.1	3.1	1.5–6.8

Model 1: crude. Model 2: age and sex. Model 3: age, sex, marital status, and educational level. Model 4: age, sex, marital status, educational level, hypertension, and diabetes.

**Table 5:** Associations of sensory impairment and frailty with improvement in subjective memory after 12 months (*n* = 1449).

Variables	*n* (%)	Model 1		Model 2		Model 3		Model 4	
		OR	95% CI	OR	95% CI	OR	95% CI	OR	95% CI
Vision									
Poor	934 (64.5)	Reference		Reference		Reference		Reference	
Robust	515 (35.5)	1.6	1.2–2.2	1.6	1.2–2.2	1.6	1.2–2.2	1.6	1.2–2.2
Hearing									
Poor	716 (49.4)	Reference		Reference		Reference		Reference	
Robust	733 (50.6)	1.5	1.1–2.0	1.5	1.1–2.1	1.5	1.1–2.1	1.5	1.1–2.1
Frailty									
Frail	298 (20.6)	Reference		Reference		Reference		Reference	
Pre-frail	717 (49.5)	2.0	1.3–3.2	2.0	1.2–3.1	2.0	1.2–3.1	2.0	1.2–3.1
Robust	434 (30.0)	2.7	1.7–4.3	2.6	1.6–4.2	2.5	1.6–4.1	2.6	1.6–4.2

Model 1: crude. Model 2: age and sex. Model 3: age, sex, marital status, and educational level. Model 4: age, sex, marital status, educational level, hypertension, and diabetes.
